# Developing optimal input design strategies in cancer systems biology with applications to microfluidic device engineering

**DOI:** 10.1186/1471-2105-10-S12-S4

**Published:** 2009-10-15

**Authors:** Filippo Menolascina, Domenico Bellomo, Thomas Maiwald, Vitoantonio Bevilacqua, Caterina Ciminelli, Angelo Paradiso, Stefania Tommasi

**Affiliations:** 1 Department of Electrical Engineering and Electronics, Technical University of Bari, Via E. Orabona 4, 70125, Bari, Italy; 2 Systems and Synthetic Biology Laboratory, Telethon Institute of Genetics and Medicine, Via Pietro Castellino 111, 80131, Naples, Italy; 3 Information and Communication Theory Group, Faculty of Electrical Engineering, Mathematics and Computer Science, Delft University of Technology, Mekelweg 4, 2628 CD, Delft, the Netherlands; 4 Department of Systems Biology, Harvard Medical School, 200 Longwood Ave, Alpert 536, Boston, MA 02115, USA; 5 Clinical Experimental Oncology Laboratory, National Cancer Institute, Via F. Hahnemann 10, 70126, Bari, Italy; 6 Bioprocess Technology Section, Department of Biotechnology, Faculty of Applied Sciences, Delft University of Technology, Julianalaan 67, 2628 BC Delft, The Netherlands; 7 Kluyver Centre for Genomics of Industrial Fermentation, Julianalaan 67, 2628 BC Delft, The Netherlands

## Abstract

**Background:**

Mechanistic models are becoming more and more popular in Systems Biology; identification and control of models underlying biochemical pathways of interest in oncology is a primary goal in this field. Unfortunately the scarce availability of data still limits our understanding of the intrinsic characteristics of complex pathologies like cancer: acquiring information for a system understanding of complex reaction networks is time consuming and expensive. Stimulus response experiments (SRE) have been used to gain a deeper insight into the details of biochemical mechanisms underlying cell life and functioning. Optimisation of the input time-profile, however, still remains a major area of research due to the complexity of the problem and its relevance for the task of information retrieval in systems biology-related experiments.

**Results:**

We have addressed the problem of quantifying the information associated to an experiment using the Fisher Information Matrix and we have proposed an optimal experimental design strategy based on evolutionary algorithm to cope with the problem of information gathering in Systems Biology. On the basis of the theoretical results obtained in the field of control systems theory, we have studied the dynamical properties of the signals to be used in cell stimulation. The results of this study have been used to develop a microfluidic device for the automation of the process of cell stimulation for system identification.

**Conclusion:**

We have applied the proposed approach to the Epidermal Growth Factor Receptor pathway and we observed that it minimises the amount of parametric uncertainty associated to the identified model. A statistical framework based on Monte-Carlo estimations of the uncertainty ellipsoid confirmed the superiority of optimally designed experiments over canonical inputs. The proposed approach can be easily extended to multiobjective formulations that can also take advantage of identifiability analysis. Moreover, the availability of fully automated microfluidic platforms explicitly developed for the task of biochemical model identification will hopefully reduce the effects of the 'data rich-data poor' paradox in Systems Biology.

## Background

Our understanding of molecular basis of complex diseases is being dramatically changed by systems investigation supported by the most advanced tools and techniques developed by the scientific community. In particular, cancer investigation has greatly benefited by systems level approaches since tumor development and progression are believed to be among those system trajectories that arise from abnormal working states. The work by Hornberg and colleagues [1] pointed out the relevance of Systems Biology approaches in the study of dynamics leading to cancer. Epidermal Growth Factor Receptor (EGFR) pathway is one of those biochemical reaction networks believed to play a central role in cancer development. As a matter of fact EGFR and receptors in the same family (ErbB2, ErbB3 and ErbB4) mediate cell to cell interactions both in organogenesis and in adult tissues [2]. The 40-year long study of this pathway led to associate overexpression of the EGFR family members to several types of cancer [3]. Because of the high clinical relevance, several efforts have been spent in the last decades in unravelling the complex dynamics of this biochemical network, as well as in finding potential targets of therapeutic intervention [4-6]. Although global models of EGFR pathway exist [7-12], many questions still remain open both in terms of model accuracy [13-15], parameter identifiability [16] and driving input design [17,18]. In this context we put the pioneering works by Arkin and colleagues [19-22], van Oudenaarden and colleagues [23] and Steuer and colleagues [24]. Other recent works have focused on the connections between optimal experimental design strategies and *structural *and *experimental identifiability *analysis of biochemical pathways; this is the case of [16,25-28].

*Structural identifiability *refers to the possibility of finding the mathematical model of the true system (see [29,30] for references in biological systems investigation), after having applied a specific search strategy in the space of the solutions.

*Experimental identifiability *[31], on the other hand, is related to the possibility of finding the mathematical representation of the true model given a predetermined set of observations. This is a central aspect of this class of identifiability problems since it is more focused on the available data and, in particular, on information content. This aspect establishes an interesting bridge between System Identification Theory and Experimental Design. The Design of Experiments (DOE) is a well developed methodology in statistics [32] focusing on the design of all information-gathering exercises where variation is present, the main objective of the whole task being the maximisation of the information obtained from experiment and the minimisation of the number of experiments. This specific task is commonly referred to as 'Optimal Experimental Design' (OED). This discipline quickly gained a significant interest among researchers mostly in natural and social sciences but became an active research field in engineering only with the pioneering work by Lennart Ljung and his standard model for dynamical system identification oriented experiments [33]. This model has been recently modified by Phair et al. [34] and Cho et al. [35].

Nevertheless the main idea behind system identification in Systems Biology remained unchanged [36]. In line with Fisher's criteria, Ljung's scheme [33] suggests to define a detailed plan of the experiments to be carried out before starting to collect input-output data from the system to be modeled. Specifications like data sampling strategies and driving inputs should be fixed in order to optimise the information yield of each experiment and to address the cost minimisation task OED is aimed at. These issues gain an even stronger relevance, if we consider the so-called 'data rich-data poor paradox' [37] resulting from the difficulties and costs involved in Systems Biology related assays. For these reasons and in order to develop a comprehensive framework for system identification in Systems Biology, we will describe how a specific issue of OED, namely Optimal Input Design (OID), can be addressed using optimality criteria and microfluidics-based experiments. As a matter of fact, microfluidic platforms have been shown to provide a powerful tool for the development of data-rich experimental strategies able to fill the gap of the previously cited paradox. Signals obtained in this stage are used as templates for the development of a microfluidic device for a flexible and automated platform for affordable single-cell experiments in Systems Biology.

In the following paragraphs, we go through a brief introduction of the EGFR model then we analyse OED and OID criteria. We review current approaches to cell stimulation in the 'Methods' section and compare them with optimality criteria derived ones. An analysis of the experimental results follows in 'Results', where we introduce a feasible design of the microfluidic device thought to speed up the process of data collection in Systems Biology by lowering the costs associated to experiments. A discussion of the results presented herein and final cues for further research are given in the last paragraph.

## Results and discussion

In order to model and understand the functionality of the EGFR signaling cascade a quantitative description of the signal dynamics is of major relevance. For this reason, we discuss the computational results obtained from *in-silico *simulations carried out using POTTERSWHEEL[38]. POTTERWHEEL is a multi-experimental fitting MATLAB package intended to allow researchers to ease model analysis and experimental setup steps. In particular, this package is one of the few in Systems Biology providing a simple interface to external input based simulation of biological pathways behavior.

To estimate the effects of different inputs on parameters estimates uncertainties, we carried out 1000 identification experiments for each of the three classes of stimuli, namely: a step input, a persistently exciting input and the time profile of the stimulation obtained from the optimisation task. Therefore we plotted a bivariate distribution of both the *V*_*max *_and *K*_*m *_of the first Michaelis-Menten based reaction ( where *v*_0 _is the initial reaction rate and [*S*] the substrate concentration, see 'Methods' for a detailed description of the mathematical modelling step) in Kholodenko's model accounting for the dephosphorylation of the EGF-EGFR dimer. It should be noted that parameter correlation can greatly affect our ability to successfully recover real parameters. This is one of the main issues arising in the field of parameter idenfitiability. In particular parameters that are structurally correlated cannot be uniquely identified from experiments. In order to investigate such peculiarities of our dynamical model we carried out an identifiability analysis based on the '*Alternating Conditional Expectation*' algorithm (*ACE*) method described in [16] and implemented in the *Mean Optimal Transformation Approach *(*MOTA*) package. We present herein the computational results so as to provide a tool for comparing the different approaches to OED in Systems Biology.

### Identifiability analysis

The statistical investigation of the properties of OED should be a primary goal when the time profile of the input is computed. Previous works in this field have focused on the comparison among alternative designs on the FIM cost values and confidence intervals [39]. Nevertheless it has been noticed that the FIM is derived from a linearisations of the least squares thus it may be unreliable in cases of considerably extended non linearities. The non identifiability of one of the parameters directly implies the functional relationships among at least two of them [16]. This phenomenon can be easily observed by plotting the joint probability distribution of each of them which will show statistically significant differences if compared with the expected multivariate normal one. From an algebraic point of view this results in the loss of rank of the covariance matrix of the multivariate distribution or, alternatively, a condition number of the same matrix asymtotically tending to infinity. Then, in order to cope with identifiability issues that may arise in identification tasks, we propose to investigate this property by using the ACE method proposed by Hengl and colleagues [16]. MOTA package is included in POTTERSWHEEL toolbox and is applied together with a linear fit sequence analysis. MOTA detects groups of two or more linearly or non-linearly related parameters. It revealed some major non-identifiabilities in the parameter space (results not shown) whose nature should be certainly investigated in order to understand their causes and possible solutions. We should remark that an integrated approach using Monte-Carlo methods for both experimental design otpimisation and parameter correlation investigation can be a feasible choice. However, we should consider that this would imply a major rise in computational costs of the approach resulting fom the high number of parameter estimation tasks to be accomplished. Not to mention the issues arising from large scale models, noise and potential multimodality that would certainly imply using a robust global optimisation algorithm.

### Monte-Carlo based analysis

**Figure 1:**
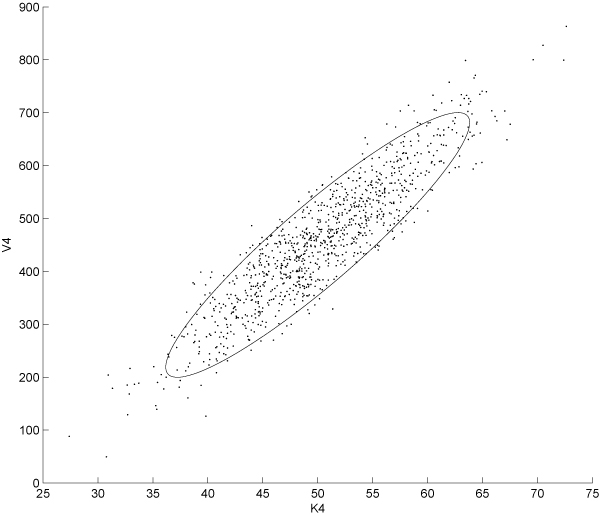
**Scatter plot of the step input-based experiment estimations**. The 95% confidence intervals for the parameters *V*_4_(on the *y *axis) and *K*_4_(on the *x *axis) in the case of estimation based on the step input driven system. Mean vector and covariance matrix are fitted on the data in order to obtain the best bivariate gaussian distribution approximating data from *in-silico *experiments.

**Figure 2:**
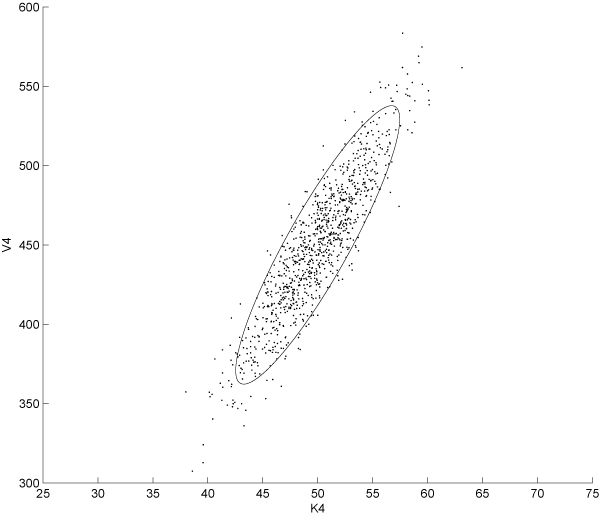
**Scatter plot of the PE input-based experiment estimations**. The 95% confidence intervals for the parameters *V*_4 _and *K*_4 _in the case of estimation based on persistently exciting input driven system. Mean vector and covariance matrix are fitted on the data in order to obtain the best bivariate gaussian distribution approximating data from *in-silico *experiments.

**Figure 3:**
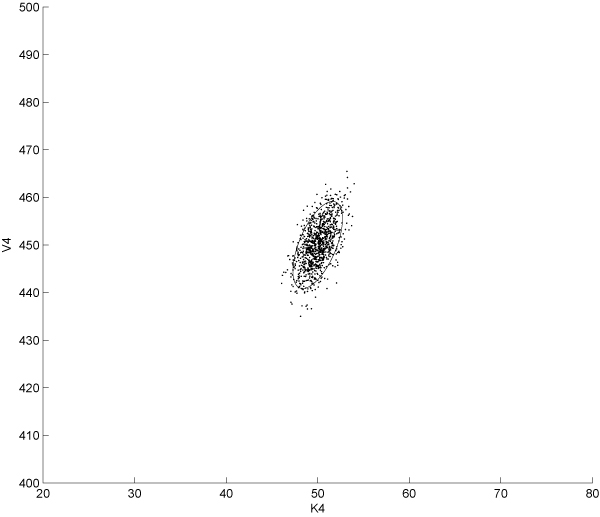
**Scatter plot of the optimal input-based experiment estimations**. The 95% confidence intervals for the parameters *V*_4 _and *K*_4 _in the case of estimation based on optimal input driven system. Mean vector and covariance matrix are fitted on the data in order to obtain the best bivariate gaussian distribution approximating data from *in-silico *experiments.

**Figure 4:**
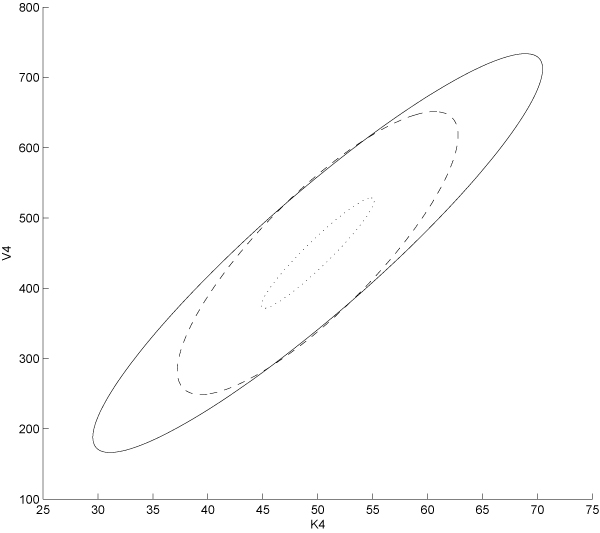
**Comparison of the 95% confidence intervals**. The three 95% confidence intervals compared; continuous line (step input), dashed line (persistently exciting input) and dotted line (optimal input). A comparison of the boundaries and positions of the ellipsoids puts in evidence that OID-based experiments are characterised by the lowest uncertainty (smallest ellipsoid area) and therefore provide the greatest amount of information on the model.

Several alternative choices to dynamic optimisation methods have been used in the context of OED, the most widely employed are direct methods such like complete parameterisation, control vector parameterisation and multiple shooting approach [39]. These methods are based on the transformation of the original infinite dimension optimisation problem into a non linear programming problem through the discretisation of the state and the stimuli or only the stimuli and the approximation of the time dependencies using locally defined function. In this work, we employed a slightly different approach with similar discretisation strategies but based on an Genetic Algorithm (GA); for this purpose we used the implementation provided by *MATLAB *through the GA routine. It should be noted that no formal proof of the convergence of GAs can be derived for the problem at hand. However, some property of this kind of approaches, just like computational complexity and efficiency can be studied in a more formal context [40]. We set the population to be composed by 200 individuals and we used the tournament system as selection criterion; crossover and mutation operators were set to 'uniform' and 'heuristic' respectively. All the other options were left at the default values while constraints on signal amplitude reflecting technological limitations were coded in the appropriate arrays (namely *A *and *b *for which *Ax *≤ *b *should be satisfied). As previously outlined the fitness function of this GA encodes for the FIM associated to the specific experimental design under investigation. In order to investigate if any statistically significant difference existed between parametric uncertainties estimated from classical and OED based experiments, we developed a Monte-Carlo based analysis with N = 1000 repetitions. We collected parameter estimates for each identification experiment. At this stage we carried out an intermediate analysis to find the parameters with the highest relative uncertainty; we selected the highest two, namely *V*_4 _and *K*_4_. A *χ*^2^goodness-of-fit test confirmed that the probability density function for these parameters can be well approximated by a normal curve. In order to compare the three experimental design selected we performed a Gaussian Mixture Model (GMM) fitting of the identified parameters starting from a bivariate distribution arising from *K*_4 _and *V*_4 _variables. Estimates were normalised and then plotted. Figure 1 shows the distribution of parameters estimates couples from sustained input experiments; Fig. 2 and 3 show the same plot for persistently exciting and OID based esperiments, respectively. The plots show the 95% confidence interval of each distribution computed as the ellipsoid centered in the mean of the bivariate distribution and having:

• major semiaxis equal to *λ*_*max*_(*Cov*)

• minor semiaxis equal to *λ*_*min*_(*Cov)*

• rotational offset with respect to the the *x *axis equal to 

where *Cov *is the covariance matrix estimated from GMM fitting and *λ*_*max*_/*λ*_*min *_its max and min eigenvalues respectively, while *y*_*eigv *_and *x*_*eigv *_are the *y *and *x *component of the eigenvectors of *Cov *matrix. Figure 4 shows the 95% ellipsoids of the three experimental designs compared. It is evident that the volume of the uncertainty ellipsoids gets minimised by more appropriate designs. Moreover the OID based strategy proves to be the one providing the best experimental conditions for accurate parameter estimation and system identification. In order to obtain a more quantitative estimation of the information gain provided by OED based experiments we performed an Ansari-Bradley test [41] on the estimated parameter values; the Ansari-Bradley test checks the hypothesis that two independent samples come from the same distribution, against the alternative that they come from distributions that have the same median and shape but different variances. Pairwise tests carried out on OID vs PE and OID vs step experiments returned p-values smaller than 0.01 thus supporting the rejection of the null hypothesis and then suggesting evidence of statistically meaningful advantages of OED based experiments over both PE and step input based ones.

## Conclusion

The intrisic quantitative nature of Systems Biology poses new issues in everyday laboratory practice. Modelling, in this context, has long suffered from data shortage; the 'data rich-data poor' paradox greatly influenced the pace towards a comprehensive understanding of molecular mechanisms governing biological systems. Nevertheless the potential of novel experimental techniques seems to promise new groundbreaking innovations thus increasing the versatility of new laboratory protocols and keeping experiment-associated costs low. Among these major limitations we should certainly mention the ability to stimulate cells in chemostats with input having very limited harmonic content. Microfluidic technology currently allows us to go beyond step like stimulation and to generate complex time-varying signals whose modulation can be achieved using control engineering strategies [42]. The availability of such tools and devices will allow us overcome the limits of indicial response for highly complex and fed-back dynamical systems identification as outlined in [43,44]. In this framework, the ability to optimally control and take advantage of the new methods and devices will be a major focus of the scientific community. This contribution presents, then, a mathematical formulation of the problem of optimal experimental design in Systems Biology by considering a case study of one of the most relevant biological pathways for cancer development. Formal derivation of problem definition results and heuristic solutions to a highly non-linear optimisation problem have been both provided. In particular we formulated the problem of OED in Systems Biology as a non-linear optimisation task in which the amount of information per experiment, quantified in the Fisher Information Matrix, is optimised by varying the stimulus time profile here representing the concentration of extracellular EGF ligand. We set up an evolutionary optimisation task aimed at finding the time sequence of the input signal that maximises the amount of information associated to the experiment. Moreover we proposed a statistical framework based on Monte-Carlo estimates for the computation of the uncertainty regions for the parameter values; identifiability analysis, on the other hand, has been carried out using the ACE approach integrated in POTTERSWHEEL package. The results shown clearly indicate that dynamic experiments outperform canonical experiments based on sustained or persistently exciting inputs. Nonetheless we should consider that the approach presented herein depends on the starting model; a sequential experimental design should be investigated in order to overcome this issue. Moreover we should consider that all the simulations reported should be validated in a series of experiments. For this reason, we proposed the microfluidic device described in the 'Methods' section. 'Labrys' goes beyond the specific context of EGFR and, associated to a Hardware Abstraction Layer like Biosteram [45], is thought to provide researchers with a fully automated platform for complex experiment development and implementation. Future work in this field will certainly require a more tight collaboration among the different competences in the field of Systems Biology aimed at the full integration of both hardware and software findings for the development of a common, powerful and versatile platform for *systems *oriented experiments.

## Methods

### Model definition

In this work, we consider the EGFR signaling network model proposed by Kholodenko and colleagues in [7]. This model explores the short term pattern of cellular responses to epidermal growth factor (EGF) in isolated hepatocytes and predicts how the cellular response is controlled by the relative levels and activity states of signaling proteins and under what conditions activation patterns are transient or sustained. BioModels database [46] provides a selection of the most common file formats of this model. For our purposes, we will use the SBML version [47] featuring 25 molecular species, 23 reactions and 50 parameters. The set of ODEs describing this model can be extracted using COPASI [48] and translated in a MATLAB SIMULINK^® ^model for further simulations and analyses. Both mass-action and Michaelis-Menten kinetics have been used by Kholodenko and colleagues, resulting in a non-linear model. In order to elicit this pathway with a driving input, we slightly modified it so as to include an external control for the ligand species, i.e. the Epidermal Growth Factor. For further experiments we selected POTTERSWHEEL[38] platform; this software provides highly powerful tools for the investigation of biological models' properties (just like parameter fitting and identifiability analysis) and, to the best of our knowledge, is one of the few ones in Systems Biology allowing researchers to easily define time evolution of forcing inputs without using complex formulations based on events and rules provided by SBML specifications.

We imported Kholodenko's model in POTTERS WHEEL and we edited the m-file so as to force EGF to be an input for our system and downstream species as our observables or outputs. This is a Single Input-Multi Output (SIMO) formulation of the EGFR and will prove to be an interesting model for the stimulation of interesting behaviors (e.g. transmission blocking zeros elicitation which is a counter-intuitive behavior of some dynamical systems which show a null output even if they are stimulated by non-trivial inputs with specific harmonic content). For our purpose, however, we will select only one downstream species to be observed just like in Single Input-Single Output systems whose study drove the field of dynamical system identification.

### Dynamical systems identification

Biological systems, just like any other dynamical system, can be described by a number of mathematical tools being ODEs an easy way to investigate systems' properties and Stochastic Differential Equations (SDE) more appropriate when small copy numbers of molecules drive system's dynamics. We will focus on the former representation approach since it's more appropriate in case of cell population-based studies (the most common ones in current practice and in many of current microfluidics-based experiments). In ODEs based models we distinguish state variables , inputs  and outputs ; from now on bold notation will be used in place of the vector one for readability sake. We define time evolution of these entities by adding to them time dependence, obtaining *x*_*i*_(*t*) *i *= 1,..., *n*, *u*_*j*_(*t*) *j *= 1,..., *m *and *y*_*k*_(*t*) *k *= 1,..., *p *respectively. We can use a set of ODEs to represent the state change through time:

(1)

Time evolution of the system state ***x***(*t*) ∈ ℝ^*n *^can be easily derived by solving the system of differential equations in Eq. 1 and imposing constraints on initial conditions ***x***(0). Notice that the rate of change of *x*_*i *_depends, in general non-linearly, on state variables *x*_*j*_, *j *= 1,..., *n*, on input trajectories *u*_*i *_and on parameters vector ***θ***.

In order to explain this representation choice we will go through a brief example. Consider a simple isomerisation reaction



if we were to model this reaction using mass-action kinetics we would model the time evolution of these species using the following set of ODEs (using *A *for the concentration [*A*])

(2)

(3)

Notice that, in order to solve these ODEs, at least two quantities are required: *k*_1 _and the inititial concentration of A species, *A*(0). The parameters vector ***θ ***is usually intended to collect these quantities.

Accurate identification of parameters governing the dynamics of biochemical reaction networks is currently considered a major challenge in Systems Biology. In fact, even though some control on initial concentration of species can be obtained with experimental protocols (e.g. starvation), rate coefficients are driven by several external factors (temperature, PH etc.) in a very complex way. Moreover accurate parameters estimation is a key step for the elicitation of interesting behaviors in cellular pathways [49].

Unfortunately the number of observable species is usually lower than the experimenter would want. For this reason we define the ***y***^*M*^(*t*, *θ*) ∈ ℝ^*p *^as the vector of measurable molecular species in an assigned experiment and we write

(4)

The observations ***y***^*O*^(*t*_*i*_) ∈ ℝ^*p *^are then given by

(5)

with

(6)

where we denoted the true parameter vector with ***θ***_0_. Here ***ε***_*i *_∈ ℝ^*p *^describes the gaussian component of the error at time *t*_*i*_. We notice that the observation function ***g***(·) together with the input function ***u***(·) and the set of sampling times ***t ***fully defines the *experimental design*. In this we glimpse the triple nature of OED which aims at establishing optimal strategies for (*a*) sampling time [50], (*b*) species to be measured [17] and (*c*) input selection [18].

### Optimal experimental design in systems biology

As previously stated OED has its main objective in maximising the information yield returned by an experiment. This is a central aspect in everyday practice in Systems Biology since experiments can be both highly expensive and time-consuming, limiting practical fasibility of otherwise promising protocols. Applications of OED in Systems Biology have been described in [17,51-55]; in particular [54,56-60] have focused on model discrimination by OED. Experimental designs are usually categorised as *starting *and *sequential designs*.

In *starting designs *no data have been previously collected and the experimenter is interested in drawing the maximum amount of information from the experiment to be planned. This is done by minimising (or maximising) a specified objective function. Within this category we identify two subcategories: *exact *and *continuous designs*.

*Exact designs *have their own objective in the optimal placement of a finite number of design points [61]; on the other hand *continuous designs *deal with the selection of a design measure, *η*, which is equivalent to a probability density over the design space.

*Sequential designs *try to develop optimal strategies for model refinement of a pre-existing model [62]. In this paper we will focus on a semi-sequential approach that can be considered a *sequential design *in that it starts from a compiled dynamic model of the EGFR pathway, but we do not use the results of this design to carry out further identification experiments since this would require non standard technological platforms. Given the potential impact of OED strategies on Systems Biology research, some researchers proposed software packages providing the user with significant opportunities for optimal experiments planning [63-65]. All of these packages are built on the principles of optimality and based on metrics being defined on the Fisher Information Matrix. This is a quite general framework for OED; unfortunately none of them currently provides a solution for Optimal Input Design. We will analyse this and other issues related to biochemical pathway stimulation in the next sections.

#### Optimal input design

Optimal Input Design for system identification provides several alternative measures of the identified model being used for optimal design [66]. Here we start by reporting some of the main results in the field [33] and then we discuss their implications in the specific case. Several contributions in this field focused on the minimisation of some measure of the variance of the estimated parameters like Fisher Information Matrix which can be used to estimate variance-uncertainty associated to parameter estimates. This process will be analysed in the next section; in this paragraph we will focus on a theoretical study of the OID for dynamic systems identification. Identification processes start with data collected on Input-Output behavior of the system under investigation.

Let the true system be described as:

(7)

for some initially unknown parameter vector *θ*_0 _∈ ℝ^*k*^, where *e*(*s*) is white noise of variance , while *G*(*s*, *θ*_0_) and *H*(*s*, *θ*_0_) are stable transfer function (a simple frequency-based representation of the input-output behavior of linear systems), with *H*(*s*, *θ*_0_) monic and minimum-phase. In most of the literature concerned with identification issues it is assumed that the system is identified with a model structure

(8)

In general if *Z*^*N *^is our source data set composed by the observation data we would want to fit these data to the model structure ℳ. ℳ describes a set of models ℳ* within which the best one is sought for. In this framework we could argue that identifiability of model structure, as it was previously defined, concerns the question whether different parameter vectors may describe the same model in the set ℳ*. However a strictly related question is whether the data set *Z*^∞ ^allows us to distinguish between different models in the set. In this context we say that a data set can be defined 'informative' if it allows us to distinguish among different models. We say that a quasi-stationary data set *Z*^∞ ^is *informative enough with respect to the model set *ℳ* if, for any two models *W*_1_(*q*) and *W*_2_(*q*) in this set:



which implies that:



for almost all *ω*. On the other hand a quasi-stationary data set *Z*^∞ ^is *informative *if it is informative enough with respect to the model set ℓ* consisting of all linear, time invariant (LTI) models. The concept of informative dataset is tightly related to concept of *persistently exciting inputs*. This can be seen easily by observing that a quasi-stationary dataset *Z*^∞ ^is *informative *if the spectrum matrix for *z*(*t*) = [*u*(*t*) *y*(*t*)]^*T *^is strictly positive definite for almost all *ω*. In fact, if we consider two models *W*_1_(*q*) and *W*_2_(*q*) and denote  = *W*_1_(*q*) - *W*_2_(*q*), applying a well known theorem on signal filtration [33], we can write:



with Φ_*x*_(*ω*) spectrum of the signal *x*(*t*) and where



Since Φ_*z*_(*ω*) is positive definite, this implies that  almost everywhere that proves the previous statement. Moreover we can observe that, given the Φ_*z*_(*ω*), for the Schur's Lemma we can assure algorithm convergence only if Φ_*u*_(*ω*) > 0 and Φ_*uu*_(*ω*) - Φ_*uy*_(*ω*) Φ_*yy*_(*ω*) Φ_*yu *_(*ω*) > 0. Evidently the only block of this array we have control on is the one representing the spectrum of input signal which directly depends on dynamical properties of the driving input signal we design. It is therefore convenient to reintroduce the concept of *persistently exciting signal of order n *for a quasi-stationary stimulus *u*(*t*): we say that a similar signal, with spectrum Φ_*u*_(*ω*) is persistently exciting of order *n *is, for all filters of the form *M*_*n *_(*q*) = *m*_1_*q*^-1 ^+ ... + *m*_*n*_*q*^*-n *^the relation  implies that . Evidently the function *M*_*n *_(*z*) *M*_*n *_(*z*^-1^) can have *n *- 1 different zeros on the unit circle (since one zero is always at the origin) taking symmetry into account. Hence *u*(*t*) is persistently exciting of *order n *if Φ_*u*_(*ω*) is different from zero on at least *n *points in the interval -*π *≤ *ω *≤ *π*. This is a direct consequence of the definition.

Signals that show such properties have been investigated and include:

• Pseudo Random Binary Signal

• Generalised Binary Noise (or Random Binary Signal)

• Sum of Sines and Filtered Noise

• Coloured Noise

Notice that this is a rather general result for the class of systems considered herein. Nevertheless a similar argumentation can be carried out by considering an information metric directly tied to experimental data and to the model to be identified. These results are commonly derived from the analysis of some metric on the Fisher Information Matrix which are commonly referred to as 'Optimality criteria'.

### Optimality criteria

As we previously outlined, we can estimate the information content of a measurement by the covariance matrix Σ of the estimated parameters. In order to illustrate how this can be done we will consider a simple estimation problem based on a widely used estimator: Maximum Likelihood Estimator (notice that these results can be extended to the Least Squares Estimator [67]). If we assume a normally distributed noise the identification process can be reduced to find ***θ ***that minimises:

(9)

The asymptotic distribution of the least squares estimate  can be computed analytically; then for a large number of observed samples the difference between real and estimated parameters tends to 0. For this reason, rather than minimising Eq. 9 directly, we linearise the function *y*_*j*_(*t*_*i*_,·) around *θ*_0 _and minimise this simpler function. Using the Taylor series expansion of  around *θ*_0 _we obtain



where Δ*θ *= ***θ***-***θ***_**0 **_and ∇ is the so called nabla differential operator defined as

(10)

and *o*(*h*^*k*^) refers to the family of functions *w*(*h*) for which

(11)

Substituting these results in the minimisation of the functional, we obtain



The minimisation of *χ*^2^(***θ***) with respect to ***θ ***brings to the following equation for the estimated deviation of the parameter vector Δ***θ***



where we identified in  the so called *Fisher Information Matrix*. Then we can solve the last equation

(12)

Therefore we can compute the covariance matrix of the parameter vector as

(13)

In order to compute this matrix we need the derivations of the observation function with respect to the parameters, ∇_***θ***_*y*_*j*_(*t*_*i*_). We then need to compute the derivative of ***g ***with respect to ***θ ***and ***x***. In addition the derivation of ∇_***θ***_*x*_*k*_(*t*_*i*_) has to be computed from the system of ordinary differential equations,

(14)

with the initial conditions (∇_***θ ***_*x*_*k*_) (0) = ∇_***θ ***_*x*_*k*_(0). Given the Fisher Information Matrix (and thus the covariance matrix of the estimated parameters) the asymptotic confidence intervals for the estimates can be computed from the multivariate normal distribution

(15)

We should also notice that, given the Cramer-Rao bound

(16)

we can easily derive a lower bound for the efficacy of a general and unbiased estimator that directly depends only on the Fisher Information Matrix. Evidently, the smaller the joint confidence intervals for the estimated parameters are, the more information the experiment carries with it. We can summarise the information about the variability in the covariance matrix into a single number by using metrics like *Det*(*F*), *max*(*λ*_*i*_) (with *λ*_*i *_representing the *i*^*th *^eigenvalue of F). This is where the alternative choice of optimality criteria arises. We can distinguish four major measures of the information content [32]:

• A-Optimal design: maximising *trace*(*F*)

• D-Optimal design: minimising *Det*(Σ)

• E-Optimal design: minimising *λ*_*max*_(Σ)

• Modified E-Optimal design: minimising 

A-Optimal designs are rarely used since they can lead to non-informative experiments [68]. D-Optimal designs can be interpreted as geometric means minimisation of the errors in the parameter estimates. E-Optimal and Modified E-Optimal designs try to minimise the largest uncertainty and the ratio of the largest and smallest uncertainties among parameter estimates respectively. Given the characteristics of each of these criteria and the computational efforts required for the specific problem we selected D-Optimality as driving criterion for our input design task.

#### Computational implementation

In order to carry out the optimisation of the input time profile we set up an optimisation routine based on a Genetic Algorithm (GA) thought to minimise an objective function encoding the D-Optimality metric on the FIM. Here we present a pseudocode of the proposed approach.

**Algorithm 1 ***Genetic_Algorithm*(*population, Fitness_Function*) **returns **individual

   **while **{*an individual has a fitness value higher*(*lower*)*than threshold*} **do**

      *new*_*population *← ∅

      **for ***i *from 1 **to ***Dim*(*population*) **do**

         *x *← *Random*_*Selection*(*population*, *Fitness*_*Function*)

         *y *← *Random*_*Selection*(*population*, *Fitness_Function*)

         *descendant *← Reproduction(*x*, *y*)

         **if ***small*_*random*_*probability *← *new*_*population ***then**

            *new*_*population *← *new*_*population *∪ *descendant*

         **end if**

         *population *← *new*_*population*

      **end for**

   **end while**

   **return ***best individual evaluated on the Fitness*_*Funtion*

**Algorithm 2 ***Reproduction*(*x*, *y*) **returns **individual

   *x *← *Length*(*x*)

   *c *← *random number in the range *{0, *n*}

   **return ***Append*(*Substring*(*x*, 1, *c*), *Substring*(*y*, *c *+ 1, *n*))

**Algorithm 3 ***Fitness Function x*(*x*) **returns **fitness value

   *input *← *x*

   *time*_*evolution *← *Simulate*(*EGFR pathway*, *input*, *θ*)

   *FIM *← *Fisher*_*Information*_*Matrix*(*time*_*evolution*)

   **return ***FIM*

Algorithm 1 shows how the optimisation task is carried out: here an individual encodes the time profile of the ligand concentration outside the cell. While the Fitness Function (FF) is used to estimate the quality of the single individual, *mutation *and *crossover *operators boost the search space exploration of the GA. This approach should help the algorithm returning the best solution (individual) to the input optimisation problem by optimising, generation after generation, the fitness value of the invididuals in the population.

### Microfluidic device design

Implementing complex time-varying signals is quite simple from a computational point of view; however obtaining realisations of signals with such properties is an active area of research in current microfluidics. Developing geometries that satisfy physical conditions for the generation of signals compliant with the specifications imposed by the theoretical results is not a trivial task. Several alternative solutions have been proposed for signal modulation in microfluidic channels [69-74] being [42,75,76] the most recent and advanced contribution in this fields; they are based on diverse physical principles like boundary diffusion controlled by relative velocity (like in H filters [74]), by exciting cells with diverse laminar flows that affect different parts of the cell etc.

In particular two main areas of research arose in this context: on the one hand interesting phenomena in fluid dynamics have been investigated [77,78] in order to address the problem of cell stimulation via contrained signals [79,80]; on the other hand the area of Digital Microfluidics has found one of its most active fields of research. In order to implement the signals obtained from the previously described input optimisation task we propose a polydimethilsiloxane (PDMS) based platform for cell stimulation which exploits the signal modulation at the microliter level. This platform has been designed to implement spectral properties of the signals that have been characterised during the theoretical study of the system under investigation: in this way it will became part of the optimal experimental design for cell stimulation.

**Figure 5:**
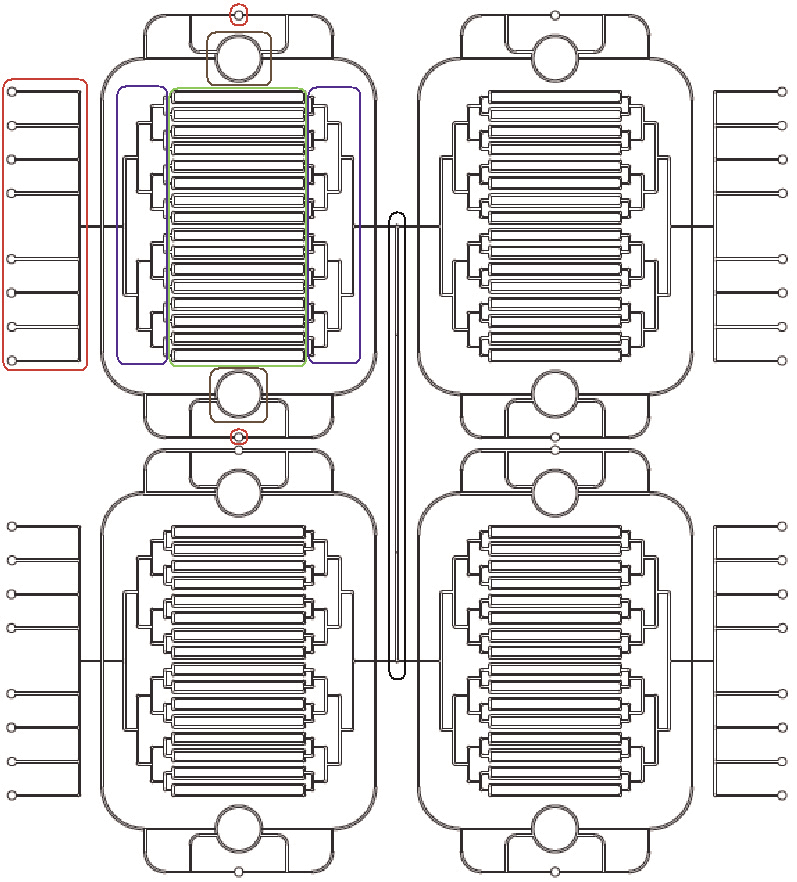
**Labrys design**. Outlook of the microfluidic device designed. The chip is composed of four functional units each featuring an input-output section (red boxes), two rotary mixers (brown box), two sets of demultiplexers and multiplexers (blue boxes) to drive the fluids in the appropriate registers (green box). The flow channel (violet box) is intended to host cells to be stimulated by compound mixes stored in the registers and moved by peristaltic valve actuation.

The platform we propose is thought to be controlled via an *Hardware Abstraction Layer *(HAL) that should allow the user to design his experiment and let the system operate with fluids and devices (like pumps, latches etc). Biostream is a suitable example of such an architecture [45]. The design of '*Labrys*', this is the name selected for the device, is reported in Fig. 5. The proposed architecture is based on the studies carried out in [42] and by Dr. Thomson [79,80]. *Labrys *features two distinct layers being the upper one in charge of the control mechanisms. This control layer has been designed using the max-flow-min-cost principle implemented in the MICADO package (v0.5) developed by Nada Amin. MICADO can also extract the microfluidic ISA the chip will be based on. The second layer has been designed using the *Mix-Store *and *Use *principle: this layer is composed of Inputs/Outputs (red boxes in Fig. 5), Muxes-Demuxes (blue boxes in Fig. 5), Registers (green boxes in Fig. 5), Mixing Units (brown boxes in Fig. 5), Flow Chamber (the 'processing unit' in violet in Fig. 5) and for this reason can be thought of as a sort of unconventional computational architecture for generating, storing and using previously characterised inputs to be used for cell stimulation. Testing such a platform, given the computational results discussed above is a simple task. A feasible approach to carry out such a process can employ an off-line generation of the discrete levels of the inducer molecule (the EGF concentration in our case) in the medium and its sequential and automated supply to the cells through a suitable valves actuation strategy.

## Competing interests

The authors declare that they have no competing interests.

## Authors' contributions

FM has conceived the idea, has designed the device, has carried out the experiments and has written the manuscript. TM and DB have partly supervised the work and assisted in manuscript preparation. VB, CC, AP and ST supervised the project and inspired the integration of the computational and device engineering sides of this work. All authors read and approved the final manuscript.

## References

[B1] Hornberg JJ, Bruggeman FJ, Westerhoff HV, Lankelma J (2006). Cancer: A Systems Biology disease. Biosystems.

[B2] Burden S, Yarden Y (1997). Neuregulins and their receptors: a versatile signaling module in organogenesis and oncogenesic. Neuron.

[B3] Yarden Y, Sliwkowski MX (2001). Untangling the ErbB signalling network. Nature Reviews Molecular Cell Biology.

[B4] Eastman A, Perez RP (2006). New targets and challenges in the molecular therapeutics of cancer. British Journal of Clinical Pharmacology.

[B5] Sebastian S, Settleman J, Reshkin SJ, Azzariti A, Bellizzi A, Paradiso A (2006). The complexity of targeting EGFR signalling in cancer: from expression to turnover. Biochimica Et Biophysica Acta.

[B6] Mendelsohn J, Baselga J (2000). The EGF receptor family as targets for cancer therapy. Oncogene.

[B7] Kholodenko BN, Demin OV, Moehren G, Hoek JB (1999). Quantification of Short Term Signaling by the Epidermal Growth Factor Receptor. J Biol Chem.

[B8] Oda K, Matsuoka Y, Funahashi A, Kitano H (2005). A comprehensive pathway map of epidermal growth factor receptor signaling. Mol Syst Biol.

[B9] Wiley HS, Shvartsman SY, Lauffenburger DA (2003). Computational modeling of the EGF-receptor system: a paradigm for systems biology. Trends in Cell Biology.

[B10] Schoeberl B, Eichler-Jonsson C, Gilles ED, Müller G (2002). Computational modeling of the dynamics of the MAP kinase cascade activated by surface and internalized EGF receptors.

[B11] Hornberg JJ, Binder B, Bruggeman FJ, Schoeberl B, Heinrich R, Westerhoff HV (2005). Control of MAPK signalling: from complexity to what really matters. Oncogene.

[B12] Breitling R, Hoeller D (2005). Current challenges in quantitative modeling of epidermal growth factor signaling. FEBS Letters.

[B13] Zhang Y, Wolf-Yadlin A, Ross PL, Pappin DJ, Rush J, Lauffenburger DA, White FM (2005). Time-resolved mass spectrometry of tyrosine phosphorylation sites in the epidermal growth factor receptor signaling network reveals dynamic modules. Molecular & Cellular Proteomics: MCP.

[B14] Olsen JV, Blagoev B, Gnad F, Macek B, Kumar C, Mortensen P, Mann M (2006). Global, in vivo, and site-specific phosphorylation dynamics in signaling networks. Cell.

[B15] Blagoev B, Kratchmarova I, Ong SE, Nielsen M, Foster LJ, Mann M (2003). A proteomics strategy to elucidate functional protein-protein interactions applied to EGF signaling. Nature Biotechnology.

[B16] Hengl S, Kreutz C, Timmer J, Maiwald T (2007). Data-based identifiability analysis of non-linear dynamical models. Bioinformatics.

[B17] Casey FP, Baird D, Feng Q, Gutenkunst RN, Waterfall JJ, Myers CR, Brown KS, Cerione RA, Sethna JP (2006). Optimal experimental design in an EGFR signaling and down-regulation model. q-bio/0610024.

[B18] Faller D, Klingmuller U, Timmer J (2003). Simulation Methods for Optimal Experimental Design in Systems Biology. SIMULATION.

[B19] Vance W, Arkin A, Ross J (2002). Determination of causal connectivities of species in reaction networks. Proc Natl Acad Sci U S A.

[B20] Samoilov M, Arkin A, Ross J (2001). On the deduction of chemical reaction pathways from measurements of time series of concentrations. Chaos (Woodbury, NY).

[B21] Vlad MO, Arkin A, Ross J (2004). Response experiments for nonlinear systems with application to reaction kinetics and genetics. Proc Natl Acad Sci U S A.

[B22] Flaherty P, Jordan MI, Arkin AP (2005). Robust design of biological experiments. Proceedings of the Neural Information Processing Symposium.

[B23] Mettetal JT, Muzzey D, Gómez-Uribe C, van Oudenaarden A (2008). The frequency dependence of osmo-adaptation in Saccharomyces cerevisiae. Science (New York, NY).

[B24] Steuer R, Kurths J, Fiehn O, Weckwerth W (2003). Observing and interpreting correlations in metabolomic networks. Bioinformatics.

[B25] Yue H, Brown M, Knowles J, Wang H, Broomhead DS, Kell DB (2006). Insights into the behaviour of systems biology models from dynamic sensitivity and identifiability analysis: a case study of an NF-[small kappa]B signalling pathway. Molecular BioSystems.

[B26] Quaiser T, Marquardt W, Monnigmann M (2007). Local identifiability analysis of large signalling pathway models. Proc of FOSBE 2007.

[B27] Anguelova M, Wennberg B (2007). Identifiability of the Time-lag Parameter in Delay Systems with Applications to Systems Biology. Proc of FOSBE 2007.

[B28] Geffen Dara, S M, A F, Findeisen Rolf, Guay M (2007). The Question of Parameter Identifiability for Biochemical Reaction Networks Considering the NF-kappaB Signal Transduction Pathway. Proc of FOSBE 2007.

[B29] Cobelli C, Romanin-Jacur G (1975). Structural identifiability of strongly connected biological compartmental systems. Med Biol Eng.

[B30] Cobelli C, Lepschy A, Romanin-Jacur G (1976). Structural identifiability of biological compartmental systems Digital computer implementation of a testing procedure.

[B31] Bellu G, Saccomani MP, Audoly S, D'Angiò L (2007). DAISY: a new software tool to test global identifiability of biological and physiological systems. Comput Methods Programs Biomed.

[B32] Pronzato L (2008). Optimal experimental design and some related control problems. 08024381.

[B33] Ljung L (1999). System Identification: Theory for the User.

[B34] Phair RD, Misteli T (2001). Kinetic modelling approaches to in vivo imaging. Nature Reviews Molecular Cell Biology.

[B35] Cho KH, Shin SY, Kolch W, Wolkenhauer O (2003). Experimental Design in Systems Biology, Based on Parameter Sensitivity Analysis Using a Monte Carlo Method: A Case Study for the TNFalpha-Mediated NF-kappa B Signal Transduction Pathway. SIMULATION.

[B36] Lipschultz CA, Li Y, Smith-Gill S (2000). Experimental design for analysis of complex kinetics using surface plasmon resonance. Methods (San Diego, Calif).

[B37] Sontag E Molecular Systems Biology and Control: A Qualitative-Quantitative Approach. Decision and Control, 2005 and 2005 European Control Conference CDC-ECC '05 44th IEEE Conference on 2005.

[B38] Maiwald T, Timmer J (2008). Dynamical Modeling and Multi-Experiment Fitting with PottersWheel. Bioinformatics.

[B39] Balsa-Canto E, Alonso A, Banga J (2008). Computational procedures for optimal experimental design in biological systems. IET Systems Biology.

[B40] Rylander BI (2001). Computational complexity and the genetic algorithm. PhD thesis.

[B41] Hollander M, Wolfe DA (1999). Nonparametric Statistical Methods.

[B42] Andrew N, Craig D, Urbanski JP, Gunawardena J, Thorsen T (2008). Microfluidic temporal cell stimulation. μTAS 08.

[B43] Kholodenko BN, Kiyatkin A, Bruggeman FJ, Sontag E, Westerhoff HV, Hoek JB (2002). Untangling the wires: a strategy to trace functional interactions in signaling and gene networks. Proceedings of the National Academy of Sciences of the United States of America.

[B44] Sontag E, Kiyatkin A, Kholodenko BN (2004). Inferring dynamic architecture of cellular networks using time series of gene expression, protein and metabolite data. Bioinformatics.

[B45] Thies W, Urbanski J, Thorsen T, Amarasinghe S (2006). Abstraction layers for scalable microfluidic biocomputing. Natural Computing.

[B46] BioModels Database. http://www.ebi.ac.uk/biomodels-main/static-pages.do?page=home.

[B47] BioModels Database – Kholodenko1999-EGFRsignaling. http://www.ebi.ac.uk/biomodels-main/publ-model.do?mid=BIOMD0000000048.

[B48] Hoops S, Sahle S, Gauges R, Lee C, Pahle J, Simus N, Singhal M, Xu L, Mendes P, Kummer U (2006). COPASI-a COmplex PAthway SImulator. Bioinformatics.

[B49] Gunawardena J (2008). Signals and Systems: Towards a Systems Biology of Signal Transduction. Proceedings of the IEEE.

[B50] Hu S (2004). Optimal time points sampling in pathway modelling. Engineering in Medicine and Biology Society, 2004 IEMBS '04 26th Annual International Conference of the IEEE.

[B51] Steinke F, Seeger M, Tsuda K (2007). Experimental design for efficient identification of gene regulatory networks using sparse Bayesian models. BMC Systems Biology.

[B52] van Riel NA (2006). Dynamic modelling and analysis of biochemical networks: mechanism-based models and model-based experiments. Brief Bioinform.

[B53] Asprey S, Macchietto S Designing robust optimal dynamic experiments 2002. http://www.ingentaconnect.com/content/els/09591524/2002/00000012/00000004/art00020.

[B54] Apgar JF, Toettcher JE, Endy D, White FM, Tidor B (2008). Stimulus Design for Model Selection and Validation in Cell Signaling. PLoS Computational Biology.

[B55] Bernaerts K, Impe JV (2005). Optimal dynamic experiment design for estimation of microbial growth kinetics at sub-optimal temperatures: Modes of implementation. Simulation Modelling Practice and Theory.

[B56] Kremling A, Fischer S, Gadkar K, Doyle FJ, Sauter T, Bullinger E, Allgower F, Gilles ED (2004). A Benchmark for Methods in Reverse Engineering and Model Discrimination: Problem Formulation and Solutions. Genome Res.

[B57] Brik Ternbach M, Bollman C, Wandrey C, Takors R (2005). Application of model discriminating experimental design for modeling and development of a fermentative fed-batch L-valine production process. Biotechnol Bioeng.

[B58] Franceschini G, Sanro Macchietto (2007). Model-based design of experiments for parameter precision: State of the art. Chemical Engineering Science.

[B59] Chen B, Asprey S (2003). On the Design of Optimally Informative Dynamic Experiments for Model Discrimination in Multiresponse Nonlinear Situations. Industrial & Engineering Chemistry Research.

[B60] Cooney M, McDonald K (1995). Optimal dynamic experiments for bioreactor model discrimination. Applied Microbiol Biotechnol.

[B61] Atkinson AC, Donev AN Optimum experimental designs. Optimum Experimental Designs.

[B62] Casey F (2006). Prediction and Optimal Experimental Design in Systems Biology. PhD in Physics.

[B63] UCSB Biosens – Bio-SPICE Dashboard. http://www.chemengr.ucsb.edu/~ceweb/faculty/doyle/biosens/BioSens.htm.

[B64] UCSB Biosens – Bio-SPICE Dashboard. http://cbbl.imim.es:8080/ByoDyn.

[B65] Gutenkunst RN, Waterfall JJ, Casey FP, Brown KS, Myers CR, Sethna JP (2007). Universally Sloppy Parameter Sensitivities in Systems Biology Models. PLoS Comput Biol.

[B66] Mehra R (1974). Optimal input signals for parameter estimation in dynamic systems-Survey and new results. Automatic Control, IEEE Transactions on.

[B67] Rodriguez-Fernandez M, Egea J, Banga J (2006). Novel metaheuristic for parameter estimation in nonlinear dynamic biological systems. BMC Bioinformatics.

[B68] Goodwin G (1987). Identification: Experiment Design. Systems and Control Encyclopedia.

[B69] Weibel DB, Whitesides GM (2006). Applications of microfluidics in chemical biology. Current Opinion in Chemical Biology.

[B70] Walker GM, Zeringue HC, Beebe DJ (2004). Microenvironment design considerations for cellular scale studies. Lab on a Chip.

[B71] Takayama S, Ostuni E, LeDuc P, Naruse K, Ingber DE, Whitesides GM (2003). Selective Chemical Treatment of Cellular Microdomains Using Multiple Laminar Streams. Chemistry & Biology.

[B72] Takayama S, Ostuni E, LeDuc P, Naruse K, Ingber DE, Whitesides GM (2001). Subcellular positioning of small molecules. Nature.

[B73] Breslauer DN, Lee PJ, Lee LP (2006). Microfluidics-based systems biology. Molecular BioSystems.

[B74] Squires TM, Quake SR (2005). Microfluidics: Fluid physics at the nanoliter scale. Reviews of Modern Physics.

[B75] Ye L, Zhang M, Alexopoulosa L, Sorger P, Jensen K (2007). Microfluidic devices for studying the response of adherent cells under short time stimuli treatment. μTAS 07.

[B76] King KR, Wang S, Jayaraman A, Yarmush ML, Toner M (2008). Microfluidic flow-encoded switching for parallel control of dynamic cellular microenvironments. Lab on a Chip.

[B77] Urbanski JP (2005). Application of Microfluidic Emulsion Technology to Biochemistry, Drug Delivery and Lab-on-a-Chip Programmability.

[B78] Thorsen T (2003). Microfluidic Technologies for High-Throughput Screening Applications.

[B79] Thomson TM (2005). TMT Thesis Project – OpenWetWare. http://openwetware.org/wiki/TMT_Thesis_Project.

[B80] Thomson TM (2005). Stimulator Project – OpenWetWare. http://openwetware.org/wiki/Stimulator.

